# A dataset for mapping the Japanese drugs to RxNorm standard concepts

**DOI:** 10.1016/j.dib.2025.111418

**Published:** 2025-02-21

**Authors:** Eizen Kimura, Yukinobu Kawakami, Shingo Inoue, Ai Okajima

**Affiliations:** a Department of Medical Informatics, Medical School of Ehime University, Toon, Ehime, Japan; b Yuimedi Inc., Tokyo, Japan

**Keywords:** RxNorm, Machine learning, Controlled terminology, Drug database

## Abstract

Observational Health Data Sciences and Informatics (OHDSI) is an international research community dedicated to large-scale observational studies using real-world healthcare data. Participation in OHDSI requires mapping local terminology systems to the OHDSI Standard Vocabulary (OSV) and transforming healthcare data into the Observational Medical Outcomes Partnership Common Data Model (OMOP CDM), a standardized database schema. Adherence to the OSV and CDM enables the integration of datasets from different countries and regions, facilitating international cross-sectional analyses and supporting the discovery of large-scale evidence and new medical knowledge.

Despite the globally recognized healthcare technology and systems excellence in Japan, Japanese real-world data (RWD) remain underutilized in international research. This is primarily due to reliance on domestically managed controlled terminologies in Japan that are not aligned with international controlled terminologies such as SNOMED CT, making mapping Japanese RWD to the CDM challenging. In addition, the wide variety of pharmaceutical products in Japan has hindered the establishment of mappings to RxNorm, the standardized drug terminology used in OHDSI.

Previously, we used a Large Language Model (LLM) to map Japanese pharmaceutical data to RxNorm. A sampling-based evaluation confirmed that the LLM could accurately identify mapping candidates. Pharmacists and a medical informatics researcher validated these mappings, resulting in an ingredient-based mapping of Japanese pharmaceutical terms to RxNorm.

Researchers interested in pharmacoepidemiology, pharmacoeconomics, and drug-related clinical decision support systems integrated with Japanese RWD can benefit significantly from this dataset. It also contains information about the target drugs, their translated names, LLM-generated suggestions, and reference data, making it suitable for developing and validating natural language processing and machine learning techniques for terminology mapping.

Specifications Table

**Table:** 

Subject	Health Informatics
Specific subject area	Mapping Japanese drug concepts to clinical drugs in RxNorm
Type of data	Table, Microsoft Excel
Data collection	We integrated the English translations of Japanese pharmaceutical information from the Drug Price Listing [1], translated using DeepL, with the ingredient names listed in JANP [2]. We embedded the Semantic Clinical Drug names from RxNorm and RxNorm Extension [3] as vector representations. Using retrieval-assisted generation, we extracted candidate RxNorm concept matches corresponding to Japanese pharmaceuticals. Then, we dynamically generated prompts to identify the final matches among these candidates, and executed these prompts with a large multimodal model (LMM) to produce mapping data between Japanese pharmaceuticals and RxNorm. [1] National Health Insurance (NHI) drug price listing code for data source for drug information in Japan [2] The Japanese Accepted Names for Pharmaceuticals database for translating drug ingredient names [3] OHDSI ATHENA for extracting RxNorm / RxNorm extension concepts included OSV.
Data source locations	[1] Ministry of Health, Labour and Welfare. National Health Insurance (NHI) drug price listing code 2024 [Internet]. Tokyo, Japan: Ministry of Health, Labour and Welfare; Available from: https://www.mhlw.go.jp/stf/seisakunitsuite/bunya/0000078916.html [2] National Institute of Health Sciences. Japanese accepted names for pharmaceuticals. Tokyo, Japan: National Institute of Health Sciences; Available from: https://jpdb.nihs.go.jp/jan [3] Odysseus Data Services. ATHENA: OHDSI Vocabularies Repository. Available from: https://athena.ohdsi.org/.
Data accessibility	Repository name: Mendeley Data Data identification number: 10.17632/y3756v8237.1 Direct URL to data: https://data.mendeley.com/datasets/y3756v8237/1
Related research article	Kimura E, Kawakami Y, Inoue S, Okajima A. Mapping Drug Terms via Integration of a Retrieval-Augmented Generation Algorithm with a Large Language Model. Healthcare Informatics Research. 2024 Oct 1;30(4):355–63.

## 1 Value of the Data

• Researchers can integrate Japanese pharmaceutical real-world data (RWD) into international cross-sectional studies.
• Researchers in medical safety, clinical decision support, pharmacovigilance, pharmacoepidemiology, or pharmacoeconomics can use this dataset to map Japanese drug RWD to standardized RWD and efficiently prepare ingredient-based analytical data.
• Researchers can use this dataset as training data or benchmarks for machine learning models in the medical domain.
• The dataset can serve as a foundation for mapping more detailed drug information, enabling analysis based on brand names, drug usage, and dosage.

## 2 Background

Observational Health Data Science and Informatics (OHDSI) conducts large-scale distributed analyses of real-world data (RWD) from healthcare institutions worldwide, encompassing patient cohorts that can include hundreds of millions of individuals [1]. These analyses provide valuable real-world evidence. Participation in OHDSI's global cross-sectional studies requires a transformation of local RWD into the relational database structure of the Common Data Model (CDM) originally developed by the Observational Medical Outcomes Partnership (OMOP) [2] and mapping of all medical terminology to the OHDSI Standard Vocabulary (OSV) [3].

Each country operates within a unique healthcare reimbursement framework, and medical terminologies in clinical information systems are typically curated independently, creating significant interoperability challenges. To address this, OSV integrates internationally controlled terminologies such as SNOMED-CT (for disease classification), LOINC (for laboratory tests), and RxNorm [4] (for standardized drug references) to create a unified vocabulary system. Consequently, institutions participating in OHDSI must map their local terminologies to OSV to ensure seamless data integration [5,6].

However, mapping medical terminologies especially those related to drug information can be extremely labour-intensive [7]. Variations in formulations, dosages, and active ingredients make it difficult to correctly align local terms with standardized OSV concepts. Although previous studies have proposed more efficient mapping methods, unresolved issues remain. Another critical issue relates to intellectual property rights: when proprietary data is used to generate mapping datasets, licensing restrictions often prevent their release as open data.

We have developed a highly accurate mapping methodology to overcome these obstacles by combining Large Language Models (LLMs) with Retrieval-Augmented Generation (RAG) [8]. Our approach relies on publicly available datasets confirmed to be free of secondary use restrictions, ensuring compliance with open data guidelines. The study showed that the Mixtral 7 × 8b model [9] provided superior accuracy compared to conventional methods. However, residual mapping errors remained, limiting the immediate usability of the resulting dataset for OHDSI research without further refinement.

To address these remaining challenges, we aimed to conduct a comprehensive final validation with two expert pharmacists, covering all oral and injectable drugs used in Japan. This step ensures that the mapping dataset meets OHDSI quality requirements and can be released as open data. In addition, we seek to identify persistent difficulties in applying machine learning-based approaches to terminology mapping and suggest directions for future methodological improvements.

## 3 Data Description

The provided dataset consists of Excel files. As shown in Table 1, the Excel sheets contain information about Japanese drugs, mapped concepts listed in the OHDSI Standard Vocabulary, and candidate mappings proposed by Mixtral 7 × 8b [9].

**Table 1:** Column names and descriptions Included in the dataset.

Column name	Description
Category	Drugs are classified into four categories: PO (“naifukuyaku”), topical (“gaiyoyaku”), dental, and injection.
NHIDPLC	National Health Insurance (NHI) drug price listing code: Drugs in the Drug Price List have been approved by the Ministry of Health, Labour and Welfare (MHLW) in Japan for use in insured medical services at healthcare facilities. Inclusion in the Drug Price List ensures that these drugs are covered by the national health insurance system when prescribed in hospitals and other medical facilities.
Ingredient	The names of drug ingredients are written in Japanese.
Specification	It specifies the active ingredient content, dosage form, and administration unit in Japanese.
Japanese Drug Name	Drug names in Japanese.
Normalized Name	Drug names were translated into English for mapping purposes.
MM8w(1-5)_STR	The strings of top five mapping candidates suggested by Mixtral 7 × 8b. If no suitable candidate is found, “N/A” is applied.
MM8w(1-5)_CUI	The concept identifier of top five mapping candidates suggested by Mixtral 7 × 8b.
MM8w(1-5)_TTY	The term type of top five mapping candidates suggested by Mixtral 7 × 8b.
OSV_ID	Mapped Concept ID listed in the OHDSI Standard Vocabulary
OSV_NAME	Mapped Concept Name listed in the OHDSI Standard Vocabulary
OSV_CLASS	Mapped Concept Class listed in the OHDSI Standard Vocabulary
COMMENT	Mapping Considerations

Note that the Ingredient, Specification, and Japanese Drug Name fields contain Japanese text strings encoded in UTF-8.

## 4 Experimental Design, Materials and Methods

### 4.1 Target

Previously, we mapped Japanese drug concepts to RxNorm using a combination of Large Language Models (LLM) and Retrieval-Augmented Generation (RAG). After generating the initial draft dataset, we curated it manually and refined it to produce the final version. For details on the method used to generate the initial draft dataset, please refer to the previous publication. This document explains the process of transforming the draft dataset into its final version.

Japanese drugs are classified into four groups: PO (naifukuyaku, oral drugs), topical (gaiyoyaku, external use drugs), dental drugs, and injections. However, as explained in the limitation section, we limited the curation to the PO and injection categories.

### 4.2 Baseline data

We derived the baseline data from the best-performing model in our previous study: LLM (Mixtral 8 × 7b) + RAG (BioBERT embedding). The LLM suggested the top five mapping candidates, which we present in columns Mixtral7×8b_STR1 to Mixtral7×8b_STR5.

### 4.3 Mapping method

Pharmacists validated the mappings by comparing the Japanese drug name, ingredient (in Japanese), specification (in Japanese), and normalized names, which they machine-translated using DeepL, with the candidate drug names suggested by the LLM. When the mappings suggested by LLM were incorrect or missing, we manually searched RxNAV [10] and ATHENA [11] for appropriate mappings. During these searches, we restricted the scope to concepts in ATHENA that met the following conditions:

DOMAIN = `DRUG'
CONCEPT = `Standard'
CLASS = (`Clinical Drug', `Clinical Drug Box', `Clinical Drug Comp', `Clinical Drug Form', `Clinical Pack', `Clinical Pack Box', `Ingredient')
VOCAB = (`RxNorm', `RxNorm Extension')
VALIDITY = `Valid'

Finally, we completed the mapping table using the concept ID, class, and name from the OHDSI standard vocabulary as references.

### 4.4 Mapping strategy for injection drugs

Injection drugs often have multiple representations, including concentration notation (w/v%) and total quantity notation (mg, g). To ensure consistency, we followed these decision steps:

Step 1. Drug name information: We examined relevant details from the drug name to identify mapping candidates.
Step 2. Specification information: We compared numerical values, such as concentration or total volume, from the specification details.
Step 3. Ratio Calculation: If neither the specification nor the drug name provided a match, we calculated the numerator/denominator ratio from the specification and searched for a candidate that produced the same result. If multiple candidates had the same concentration, we selected the one whose numerator/denominator representation was the closest match.

Priority order:

When both the total and concentration could be interpreted, we prioritized total.
We prioritized mappings to concepts included in RxNorm to ensure alignment with standardized terminology. If no valid entry existed in RxNorm, we selected suitable candidates from the RxNorm extension.

Example 1:

Drug Name: acelio Bag for intravenous injection 1000 mg (in Japanese)
Ingredient: Acetaminophen
Specification: 1000 mg / 100 mL (1 bag)
Mapping: We chose Acetaminophen 1000 MG Injectable Solution over Acetaminophen 100 MG/ML Injectable Solution because the drug name emphasizes the total amount of 1000 mg per unit.

Example 2:

Drug Name: RADICUT injection 30 mg (in Japanese)
Ingredient: Edaravone
Specification: 30 mg / 20 mL (1 vial)
Mapping: Since the Edaravone 30 MG Injectable Solution was not registered in RxNorm, we used the concentration information (30 mg / 20 mL = 1.5 mg/mL) to select the Edaravone 1.5 MG/ML Injectable Solution.

Example 3:

Drug Name: Docetaxel I.V. Infusion 20 mg/1 mL “Chemiphar” (in Japanese)
Ingredient: Docetaxel
Specification: 20 mg / 1 mL (1 vial)
Mapping: Based on the concentration notation, we selected Docetaxel 20 MG/ML Injection as the primary choice. However, to distinguish it from other specifications (e.g., 80 mg/4 mL), we mapped it to Docetaxel 20 MG Injection*.*

## Limitations

The mapping of these drugs is based on their active ingredients. Currently, target mappings are performed against standard concepts included in the OHDSI Standard Vocabulary. As a result, the following limitations exist:

Due to limited researcher resources and the anticipated low demand for RWD analysis, mappings are not provided for the Topical and Dental categories, which often include products unique to Japan.

Drugs containing multiple active ingredients or those where multiple standard concepts could serve as mapping targets, such as Chinese medicines and traditional Japanese herbal medicines (kampo medicine), and infusions, are not mapped.

For injectable drugs, various notations are used, including concentration (w/v%) and total content (mg or g), resulting in multiple potential concepts for mapping. Therefore, the criteria described in the Method section have been applied. While this does not pose an issue for ingredient-based analyses, researchers should carefully consider dosage and unit conversions when conducting analyses where the administered dose is critical*.*

## Ethics Statement

The project does not involve human studies, animal studies, data collected through social media, or data containing personal information. Therefore, ethical approval was not sought. We can confirm that this manuscript conforms to the standards of ethics in publishing*.*

## CRediT Author Statement

**Eizen Kimura:** Conceptualization, Methodology, Software, Formal analysis, Project administration, Funding acquisition, Writing - Original Draft. **Yukinobu Kawakami:** Validation, Investigation, Data Curation. **Shingo Inoue:** Validation, Data Curation. **Ai Okajima:** Validation, Investigation, Data Curation.

## Data Availability

Mendeley DataMapping dataset between Japanese Pharmaceuticals and RxNorm (Original data).
